# Nanocomposite Photoanisotropic Materials for Applications in Polarization Holography and Photonics

**DOI:** 10.3390/nano13222946

**Published:** 2023-11-14

**Authors:** Dimana Nazarova, Lian Nedelchev, Nataliya Berberova-Buhova, Georgi Mateev

**Affiliations:** 1 Institute of Optical Materials and Technologies, Bulgarian Academy of Sciences, 1113 Sofia, Bulgaria; dimana@iomt.bas.bg (D.N.); nberberova@iomt.bas.bg (N.B.-B.); georgi@iomt.bas.bg (G.M.); 2 Department of Physics, University of Chemical Technology and Metallurgy, 1756 Sofia, Bulgaria

**Keywords:** nanocomposite materials, nanoparticles, photoanisotropic materials, azopolymers, polarization holographic gratings, surface relief gratings

## Abstract

Photoanisotropic materials, in particular azodyes and azopolymers, have attracted significant research interest in the last decades. This is due to their applications in polarization holography and 4G optics, enabling polarization-selective diffractive optical elements with unique properties, including circular polarization beam-splitters, polarization-selective bifocal lenses, and many others. Numerous methods have been applied to increase the photoinduced birefringence of these materials, and as a result, to obtain polarization holographic elements with a high diffraction efficiency. Recently, a new approach has emerged that has been extensively studied by many research groups, namely doping azobenzene-containing materials with nanoparticles with various compositions, sizes, and morphologies. The resulting nanocomposites have shown significant enhancement in their photoanisotropic response, including increased photoinduced birefringence, leading to a higher diffraction efficiency and a larger surface relief modulation in the case of polarization holographic recordings. This review aims to cover the most important achievements in this new but fast-growing field of research and to present an extensive comparative analysis of the result, reported by many research groups during the last two decades. Different hypotheses to explain the mechanism of photoanisotropy enhancement in these nanocomposites are also discussed. Finally, we present our vision for the future development of this scientific field and outline its potential applications in advanced photonics technologies.

## 1. Introduction

The demand for new materials to develop modern technologies is constantly growing. Although nanocomposite (NC) materials have been in use for a long time, they are constantly being improved to enable increasingly advanced applications. The new materials, obtained by combining two or more materials on nanoscale level, in most cases not only combine the qualities of their components, but also yield new, advantageous properties. In many cases, the nanoparticles (NPs) incorporated in nanocomposite materials modify and improve the optical, mechanical, and electrical properties of the matrix.

The optical properties of nanocomposite materials have been used since ancient times. Stained glass windows and ancient works of art exemplify their applications. A popular example of the intriguing optical features of nanocomposites is the Lycurgus cup from the 4th century AD [1]. In this case, the basis is the effect obtained from the excitation of the surface plasmon resonance due to the presence of gold and silver nanoparticles in the glass, which is expressed in a change in the color of the cup depending on whether it is viewed in transmission or reflection.

The combination of organic and inorganic materials in nanocomposites opens up a new and exciting area in applied optics. Inorganic components modify the optical properties of the organic components. In addition, the organic matrix may provide a flexible and ordered structure to the nanocomposite materials. For example, polymers can be hybridized with nanoparticles, modifying the refractive index, birefringence, and other characteristics of the composite material [2,3,4,5,6,7]. 

From the point of view of holographic applications, in recent years, special attention has been paid to nanocomposite materials composed of photopolymers and nanoparticles [8,9,10,11,12,13,14,15,16,17,18,19,20,21,22,23,24,25,26,27,28,29,30,31,32,33,34,35,36]. The ability of these materials to modify their structure and surface through light-induced polymerization is used. Problems like the creation of practically non-shrinkable holographic recording media have been solved by using various types of nanoparticles. Many applications of these materials in holography have been outlined by Tomita et al. in their topical review on photopolymerizable nanocomposite photonic materials [12]. The modern trends in the development of light-sensitive media for holography applications are presented in a review article by Barachevsky [37]. Other type of hybrid organic/inorganic (or nanocomposite) materials include polymer-dispersed liquid crystal (PDLC) materials or liquid crystals doped with nanoparticles of photosensitive polymers, which have been intensively studied by many research groups [38,39,40,41,42,43,44,45,46,47,48,49,50,51,52,53]. However, PDLCs are outside the scope of the present review, as they cover a very wide area of research that requires a separate consideration and analysis.

In this paper, we will put a special focus on azopolymer-based nanocomposites, which are applicable in polarization holography. They most often represent an azopolymer matrix doped with metallic or non-metallic nanoparticles. In order to use a given material for polarization holographic recording, it must be photoanisotropic, or in other words, it must be able to register and record the polarization of light. The higher the value of the photoinduced birefringence, the greater the diffraction efficiency of the polarization diffraction gratings recorded in this material. Azobenzene-containing materials are often the preferred recording media for polarization holography due to their high values of photoinduced birefringence, allowing for the inscription of highly efficient polarization-selective holographic gratings. These gratings have specific polarization properties [54,55,56,57] that enable various applications in the fields of polarization-selective optical elements, high-capacity data storage, and many others [58,59,60,61,62]. A polarization diffraction grating is the key component in the design of a spectrophotopolarimeter, which can measure simultaneously in real time the spectra of all four Stokes parameters of light, as reported by Todorov and Nikolova [58,60]. Provenzano et al. reported the application of anisotropic gratings for circular dichroism measurements using a new configuration that is simpler than the conventional one [63]. Particularly important are the applications of polarization holographic gratings as polarization-selective diffractive optical elements (PSDOEs). Specially designed gratings can be used as circular polarization beam splitters [64] or to convert a circularly polarized incident beam into a linearly polarized beam [65]. They can act as bifocal, spherical, cylindrical, and tunable Fresnel lenses or microlens arrays [66,67,68,69], or they can be designed to generate asymmetric diffraction [70]. 

The implementation of high-density and high-capacity volumetric data storage is another important application of photoanisotropic materials. Polarization holography enables polarization multiplexing, as shown by Nikolova and Ramanujam [55,71]. Lin et al. designed and experimentally demonstrated a polarization multiplexing holographic memory with an increased storage capacity using a circular polarization recording configuration [72]. Similarly, polarization holographic gratings were applied for polarization multichannel multiplexing, vector beam storage, and fabrication of polarization multiplexing diffracting optical elements [73], self-interference incoherent digital holography [74,75], virtual reality displays [76,77], and also 4G optical elements with spatially modulated birefringence across the surface [78,79]. Yang et al. and Xia et al. applied the diffraction characteristics of anisotropic gratings to demonstrate logic operations using Boolean algebra for all-optical diffraction elements using an azo-dye doped polymer film [80,81].

In 1995, Rochon et al. [82] and Kim et al. [83] first reported a very important effect in azopolymers: the formation of surface relief during polarization holographic recordings. They found that in this case together with the polarization grating in the volume of the media, a surface relief grating (SRG) is formed in the azobenzene-containing polymer films. The surface relief grating is produced by an interference pattern of light and is due to a photoinduced mass transport of the azopolymer. Since SRG formation is an effective and simple nanofabrication process, it has provoked significant interest in many research areas [84,85,86,87,88,89,90]. Various applications based on its unique features have been reported so far in optics [12,28,70,91,92,93], sensors [24,88,94,95,96,97,98], mechanical applications [99,100,101,102,103], optically controlled alignment [104,105,106], etc.

The development of azobenzene-containing nanoparticles and the possibility for optical manipulation of their shape and size also opens several new fields of applications [107,108,109,110]. A very significant elongation of the azopolymer-containing drops has been observed when illuminated with linearly polarized light—up to six times their initial diameter [109]. It has also been demonstrated that their shape can be controlled by the direction of light polarization and the laser irradiation time. Potential applications in optical signal control as well as mechanical motion control of photosensitive soft materials at the microscale and nanoscale were also suggested [110].

All these applications require an efficient photoanisotropic media with high photoinduced birefringence. To date, photoinduced anisotropy has been observed in various photosensitive materials. Amongst them, the azobenzene-containing materials have been extensively used due to the high values of photoinduced birefringence and diffraction efficiency achieved in them when recording polarization diffraction gratings [111,112,113,114,115,116,117,118]. Under irradiation with polarized light within their absorbance band, the azobenzene groups undergo a series of *trans-cis-trans* photoisomerizations until they reorient perpendicularly to the polarization of the pump light. This anisotropic orientation of the azobenzene groups leads to birefringence, which furthermore can be spatially controlled with high resolution over the area of the optical element. A probe beam, with a wavelength outside of the absorption band of the used material, can non-destructively read this birefringence. Diverse applications have inspired the investigation of large number of azopolymers [117,118,119,120]. A commercially available azopolymer, commonly denoted as PAZO (poly[1-4-(3-carboxy-4-hydrophenylazo)benzensulfonamido]-1,2-ethanediyl, sodium salt]), is often the preferred material for polarization holography by many research groups due to the high values of photoinduced birefringence and large amplitude of the surface relief gratings inscribed in it [121,122,123,124,125,126,127,128,129,130,131,132]. In addition, its solubility in water and methanol allows for the easy preparation of nanocomposite materials using water suspensions of nanoparticles, for example gold or silver NPs. 

Many approaches have been implemented in order to obtain polarization-sensitive materials with the highest possible photoinduced birefringence. Most often, new azopolymer architectures have been synthesized and tested with various substituents for the azochromophores, different spacer length between the main and the side chain of the azopolymer, etc. Alternatively, some methods are aimed at improving the performance of already existing azopolymers, for example, via thermally assisted recording [133], or by doping the azopolymer matrix with nanoparticles, which is the main subject of this review.

We present an overview of the polarization-sensitive nanocomposite materials with an azobenzene-containing matrix doped with nanoparticles with different compositions, concentrations, shape and sizes. As will be shown, these photoanisotropic nanocomposite materials stand out with their improved optical properties, like higher birefringence, faster response, and better stability. We examine the key contributions of each work and explore the ideas that have been suggested to improve the different polarization-sensitive azopolymer materials.

This review is organized as follows: In the Introduction so far, we briefly explored the various applications of polarization holography and azobenzene-containing photoanisotropic materials, which determine the need for further development of polarization sensitive materials and increasing their photoinduced anisotropy. In Section 2, we present the most commonly used components of nanocomposite photoanisotropic materials (NPMs). The first of the two subsections is dedicated to the nanoparticles used for doping the NPM and the second to the azo-containing matrices used. In Section 3, the studies of the different groups of researchers who work in the field of nanocomposite photoanisotropic materials are classified according to the composition of the nanoparticles. This section is again divided into two subsections: the first is dedicated to NPM containing non-metallic nanoparticles, and the second to NPM containing metallic NPs. Section 4 presents research on the influence of the size and shape of the nanoparticles on the increase in the photoinduced anisotropy of the materials. Accordingly, both Section 3 and Section 4 examine the resulting impact on the photoinduced birefringence or absorption changes of the nanocomposite materials in terms of the optimal nanoparticle concentrations. In Section 5, the diffraction efficiency and surface relief height of recorded polarization holographic gratings in these materials are analyzed. Finally, in Section 6 and Section 7, we include a comparative table to summarize the optical properties of a large number of NPMs, a discussion about the mechanism of enhancement of the optical properties of the nanocomposites due to the presence of nanoparticles in azobenzene-containing materials, conclusions, and ideas for future studies.

## 2. Components of the Nanocomposite Photoanisotropic Materials

In this section we will briefly introduce the two most important components of the nanocomposite photoanisotropic materials: (i) nanoparticle dopants with various chemical compositions, sizes, and morphologies, and (ii) photoanisotropic matrices based on azodyes or azopolymers. Due to their nanoscale dimensions, the nanoparticles are usually characterized using transmission electron microscopy (TEM) or scanning electron microscopy (SEM).

Furthermore, these techniques allow us to determine the size distribution of the NPs and also the way they are dispersed within the nanocomposite thin film samples. The azobenzene-containing NC components (azodyes or azopolymers) are presented with their chemical structures that give the essential information about the azochromophores, the substituents used, the length of the side-chain spacer, etc.

### 2.1. Nanoparticles

**Figure 1:**
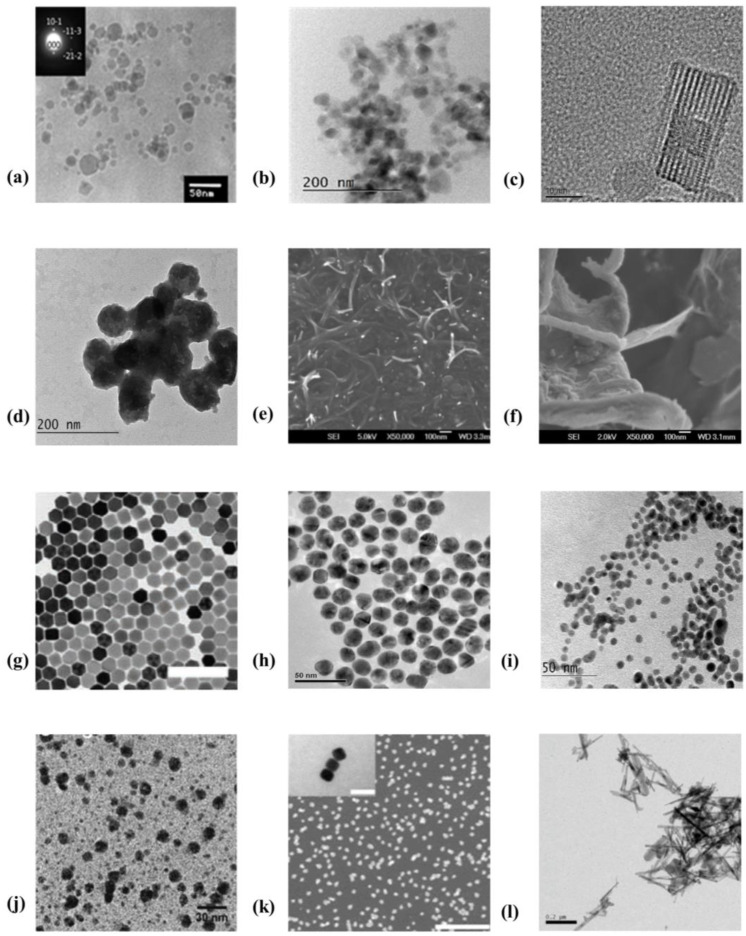
TEM/SEM images of NPs with different composition and shape: (**a**) TEM image of the nanocomposite film made from the copolymer P_1–2_ doped with ZnO nanoparticles. In the inset is shown the selected area electron diffraction image (reprinted with permission from [139]); (**b**) TEM image of TiO_2_ nanoparticles in PAZO thin film (reprinted with permission from [148]); (**c**) TEM image of a MFI zeolite rectangular nanoparticle (reprinted with permission from [151]); (**d**) TEM image of the chalcogenide NPs (GeTe_4_)_100−x_Cu_x_ (reprinted with permission from [164]); (**e**) SEM image of carbon nanotubes (reprinted with permission from [137]); (**f**) SEM image of carbon nanofibers (reprinted with permission from [137]); (**g**) TEM image of upconverting hexagonal nanoparticles (scale bar is 200 nm) (reprinted with permission from [152]); (**h**) TEM image of Au colloidal solution (reprinted with permission from [160]); (**i**) TEM image of Au NPs (reprinted with permission from [156]); (**j**) TEM image of Ag (reprinted with permission from [155]); (**k**) SEM image of Ag nanocubes. The inset shows a TEM image of silver nanocubes (scale bar is 100 nm) (reprinted with permission from [165]); (**l**) TEM image of goethite nanorods (reprinted with permission from [150]).

Nanoparticles with different compositions have attracted the attention of researchers as possible dopant components, such as quantum dots (QDs) [134,135,136], carbon nanotubes (CNTs), and carbon nanofibers (CNFs) [137,138], ZnO [128,139,140,141,142], SiO_2_ [143,144], TiO_2_ [145,146,147,148], semiconductor nanoparticles like tellurium containing chalcogenide system (GeTe_4_)_100−x_Cu_x_ [149] and goethite (α-FeOOH) nanorods [129,150], nanozeolites [151], and upconverting nanoparticles (UCNPs) [152]. Gold (Au) and silver (Ag) NPs are the most commonly used metallic nanoparticles [132,146,153,154,155,156,157,158,159,160,161]. Studies have also been conducted with bioactive metals, such as copper and nickel (Cu, Ni) [162,163]. Nanocomposites with various sized nanoparticles have also been investigated. Very small nanoparticles with sizes in the range 2.5–10 nm have been used by some researchers. There are more studies with medium-sized NPs from 10 nm to 50 nm, whereas fewer studies focus on the larger NPs with sizes in the range 150–600 nm. Nanoparticles of different shapes have also been used. The most commonly used shape is spherical. There are also several studies of nanorods, as well as of nanoparticles with hexagonal, cubic, or rectangular shape. Examples of these different types of nanoparticles are presented graphically in Figure 1.

### 2.2. Commonly Used Azo-Containing Matrices for Nanocomposites

**Figure 2:**
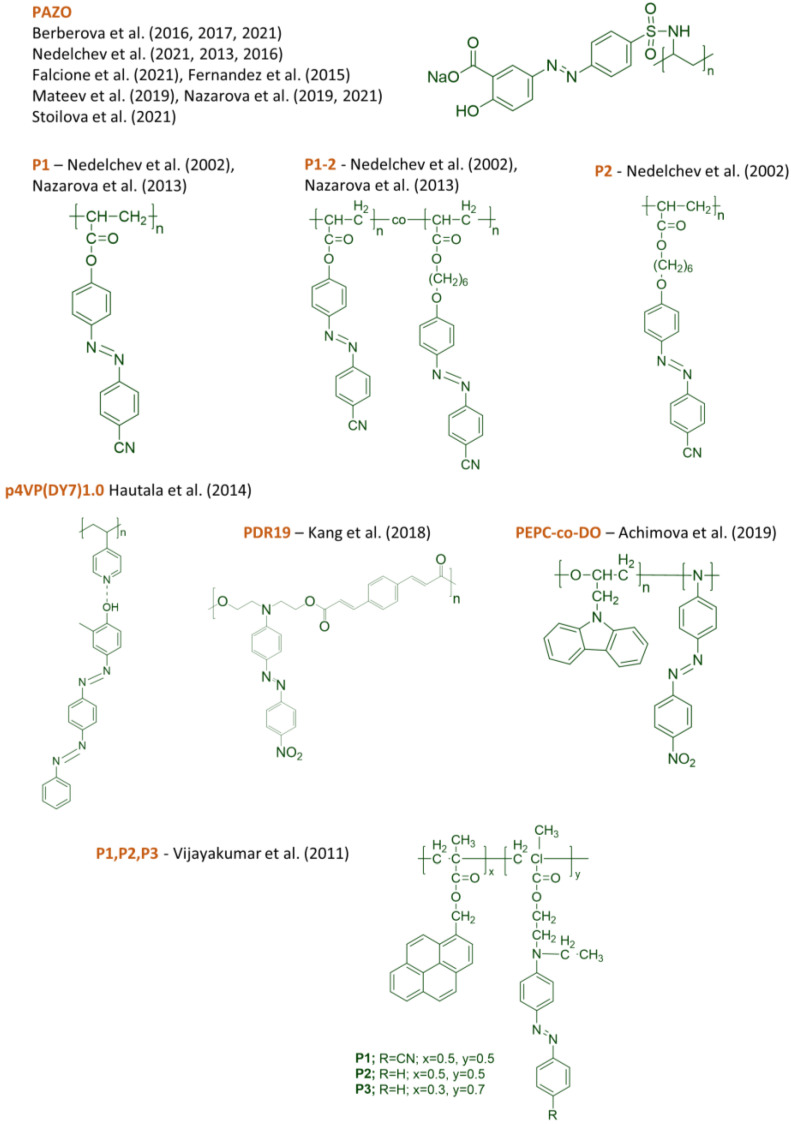
Chemical structures of some azopolymers used as NPM components in the studies of Berberova et al. [128,141,158], Nedelchev et al. [129,139,140,150], Falcione et al. [132], Fernandez et al. [146], Mateev et al. [147,162], Nazarova et al. [148,156,166], Stoilova et al. [149,163], Hautala et al. [161], Kang et al. [145], Achimova et al. [135], and Vijayakumar et al. [167].

The chemical structures of some azo-containing polymers used for nanocomposite components are presented in Figure 2. A very commonly used azopolymer, as we have already noted, is the commercially available azopolymer PAZO (poly[1-4-(3-carboxy-4-hydrophenylazo)benzensulfonamido]-1,2-ethanediyl, sodium salt]). PAZO was used as the base to obtain photoanisotropic NCs by many authors, like Berberova et al. [128,141,158], Nedelchev et al. [129,140,150], Falcione et al. [132], Fernandez et al. [146], Mateev et al. [147,162], Nazarova et al. [148,156], Stoilova et al. [149,163], and others.

Some other azopolymers have also been employed as components of NPM and are denoted by the authors as follows: P_1_—Nedelchev et al. [139], Nazarova et al. [166], P_1–2_—Nedelchev et al. [139], Nazarova et al. [166], P_2_—Nedelchev et al. [139], p4VP(DY7)_1.0_ Hautala et al. [161], PDR19—Kang et al. [145], PEPC-co-DO—Achimova et al. [135] and P1, P2, P3—Vijayakumar et al. [167].

**Figure 3:**
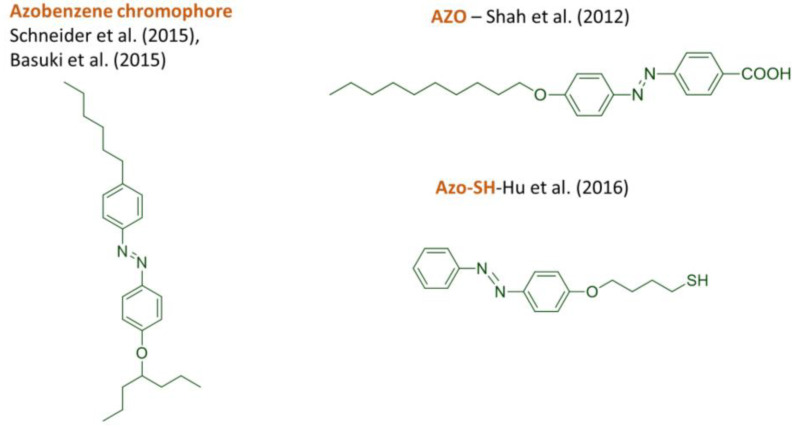
Chemical structures of some azodyes used as components of the nanocomposite photoanisotropic materials investigated by Schneider et al. [168], Basuki et al. [169], Shah et al. [142], and Hu et al. [170].

In other studies, nanocomposites are composed from photoanisotropic azo dyes, nanoparticles and a non-photoanisotropic polymer matrix [142,168,169,170,171]. The chemical structures of some of the azo dyes used in these cases are shown in Figure 3.

### 2.3. Main Optical Parameters of the Nanocomposite Photoanisotropic Materials

As was already mentioned in the Introduction, an essential parameter that characterizes the optical response of any photoanisotropic media, including the NPM, is the maximal value of the photoinduced birefringence, denoted as Δ*n*_max_. It is easily measured by a simple *pump-probe* optical scheme, which does not require coherent lasers or vibration isolation, and for this reason it is the most commonly used parameter to evaluate the enhancement of the nanocomposites’ performance in comparison with the non-doped azobenzene-containing material.

In case of polarization holographic recording, two more parameters are used to quantify the behavior of the nanocomposite photoanisotropic materials, namely the diffraction efficiency (DE) and height of the formed surface relief grating (*h*_SRG_).

These three main parameters are studied and compared throughout the present review to demonstrate the effect of adding different nanoparticles to the azodye/azopolymer matrix.

## 3. Nanocomposite Photoanisotropic Materials Doped with Nanoparticles with Different Compositions

Nanocomposite photoanisotropic materials are presented in this Section in accordance with the composition of the nanoparticles used as their components. The nanoparticles themselves are grouped into two main subdivisions: (i) non-metallic NPs, which include quantum dots, carbon nanotubes, carbon nanofibers, ZnO, SiO_2_, TiO_2_, semiconductors, zeolite, and other NPs, and (ii) metallic NPs, which also include metal complexes.

### 3.1. NPM Doped with Non-Metallic Nanoparticles

#### 3.1.1. Quantum Dots (QDs), Carbon Nanotubes (CNTs), and Carbon Nanofibers (CNF)

Quantum dots (QDs) are nanoparticles with dimensions usually smaller than the exciton Bohr radius for the given material, so their properties are determined by their quantum effects. They have been widely used recently and they are particularly promising for nanocomposite photoanisotropic materials applications as a dopant due to their amazing optical properties such as the size-dependent broad tunability of absorption and emission bands [171,172]. Larger QDs have a greater spectrum shift toward red compared to smaller QDs.

Carbon nanotubes (CNTs) and carbon nanofibers (CNFs) are large molecules comprised of hybridized carbon atoms in hexagonal arrangement, which can be divided into single-walled carbon nanotubes (SWCNTs) formed by a single sheet of graphene and multiwalled carbon nanotubes (MWCNTs) formed by rolling up multiple sheets of graphene. Carbon nanotubes enable conductivity in nanocomposite materials and retain their durability and features. One of the unique properties of the CNTs and CNFs is the possibility of fine-tuning the wavelength selectivity of emission and detection of light through the nanotube structure [137,138].

In 2008, Li et al. studied the enhancement of three-dimensional optical memory using quantum dots and azo-dye doped polymers [136]. They studied the azo-dye Disperse Red 1 (DR1) and cadmium sulfide (CdS) QDs with three different sizes in chloroform solution labeled as CdS 366 (2.43 nm), CdS 433 (4.57 nm), and CdS 441 (4.89 nm), respectively. The dynamic evolution of the absorbance change in DR1 was monitored and it was observed that there is no isomerization enhancement of DR1 doped with CdS 366 as the absorbance decreases approximately by the same extent as for DR1 itself in both polarization directions. However, a remarkable enhancement in global absorbance of the CdS 441 and CdS 433 dispersed samples was observed.

In 2019, QDs of the composition Cd_0.2_Zn_0.8_Se were doped in the copolymer PEPC-co-DO (polymeric matrix of poly-epoxypropylcarbazole modified with azo-dye Disperse Orange 3) and the prepared thin film was studied [134,135]. Achimova et al. [135] and Loşmanskii et al. [134] observed that the difference between the amplitude and the phase grating is more pronounced for PEPC-co-DO with Cd_0.2_Zn_0.8_Se QDs thin films than for non-doped PEPC-co-DO thin films. On the other hand, they did not observe an increase in the diffraction efficiency or surface relief of the nanocomposite films.

Another type of nanoparticle that has been studied for improving the photoinduced properties of azocompounds is carbon nanotubes (CNTs) [137,138]. Various strategies have been used by researchers for synthesizing azocompounds and CNT nanocomposites, e.g., the azopolymer can be functionalized [167], or the azobenzene chromophore can interact with the CNTs through chemical bonds, which are strong enough when the trans-conformer is parallel to the long axis of the nanotubes [173,174]; alternatively, the nanocomposites can be prepared via in situ doping [175,176].

Rodríguez-González et al. [137] demonstrated that a small amount (0.5 wt. %) of CNTs or CNFs has a positive impact on the photoinduced birefringence of a liquid crystal azopolymer (P0C6). They achieved a maximum increase in the photoinduced birefringence value by in situ doping this azopolymer with carbon nanofibers. They also demonstrated that the different doping methods determine the different way the azopolymer relaxes. The mixing method displays a typical relaxation, while the in situ method displays an inverse relaxation.

Díaz-Constanzo et al. [138] also reported an enhancement of the optical response in a biodegradable polymer/azo-dye (PLA/DO3) film by adding multi-walled carbon nanotubes. They studied this effect at different temperatures and concluded that the enhancement occurs in the entire temperature range. At room temperature they achieved optical anisotropy for the nanocomposite 100% larger than the one obtained for the material without carbon nanotubes.

In 2011, Vijayakumar et al. [167] studied azobenzene-derived photoactive polymers (P1, P2 and P3) containing pyrene pendants designed and synthesized for the noncovalent functionalization of single-walled carbon nanotubes. The thermal stability of the polymers was found to be higher in the composite state; the birefringence was also increased.

Using azobenzene derivatives as photoswitchable molecules, Schneider et al. [168] demonstrated light-induced conductance switching in carbon nanotube–polymer composites with chromophores exhibiting no conductance. No decrease in the switching amplitude was observed from cycle to cycle. It was suggested that the azobenzene moieties act as trap centers. An enhanced switching amplitude in MWCNT-polymer nanocomposites containing azobenzene-based chromophoresis was also reported by Basuki et al. [169].

Although apparently the CNTs are good dopants for increasing birefringence of azopolymers, the main disadvantage of CNTs, which limits their wide application, is their high cost.

#### 3.1.2. Zinc Oxide (ZnO) Nanoparticles

One of the most commonly used nanostructures recently are the zinc oxide (ZnO) nanostructures. The interest in them has increased drastically [177,178,179,180,181,182,183]. Intense research by many different groups has focused on novel nanostructures with different shapes ranging from nanospheres and nanowires to nanorods and other more complex shapes. ZnO is a direct wide bandgap material with a large exciton binding energy. This makes it an attractive optical material based on exciton recombination at room temperature or even higher.

In 2012, the group of Nazarova and Nedelchev started a series of studies on photoanisotropic nanocomposites, more specifically azopolymers doped with different nanoparticles. The experimental results demonstrate that these nanocomposites have enhanced photoanisotropic properties. In the first study of these series, three azopolymers synthesized by our group were used [139]. The amorphous azopolymer P_1_, the amorphous azo copolymer P_1–2_, and the liquid crystalline azopolymer P_2_ were doped with ZnO nanoparticles with size < 50 nm and different concentrations. A significant increase in the photoinduced birefringence in the nanocomposite films of the azopolymers doped with ZnO nanoparticles compared with samples made from non-doped azopolymers was observed [139,140]. This increase was up to 47% for the azopolymer P_1_ and concentration of the nanoparticles 0.5%. An improvement in the response time of more than 25% for some of the nanocomposites was also observed.

This study was continued and expanded using a different azopolymer—the commercially available azopolymer PAZO (Poly[1-[4-(3-carboxy-4-hydroxyphenylazo) benzenesulfonamido]-1,2-ethanediyl, sodium salt]) in a hybrid PAZO-based organic/inorganic material with incorporated ZnO nanoparticles [128]. The enhancement of the photoinduced birefringence in the nanocomposite films compared with the samples made from non-doped azopolymers was confirmed. For this system, a maximum increase in the birefringence was reached for a 10% concentration of NPs. The spectral dependence of the birefringence in time for these NCs was also investigated and the optimal wavelength for recording was established [184].

Later, the same nanocomposite material (PAZO doped with ZnO NPs) was used to record polarization holographic gratings and the experimental results indicate that both the diffraction efficiency and the height of the surface relief grating for the nanocomposite samples were enhanced as well [141]. The height of the relief was increased with 9 nm for the grating with spatial frequency 625 lines/mm and with 42 nm for spatial frequency 1250 lines/mm. The diffraction efficiency was also enhanced with 20% for the 625 lines/mm grating and twice for the 1250 lines/mm grating. The reported successful realizations of holographic diffraction gratings with enhanced surface relief recorded in NPM are discussed in more detail in Section 5.

Again in 2012, Shah et al. [142] reported the synthesis and spectroscopic characterization of nanohybrid structures consisting of an azobenzene compound grafted on the surface of zinc oxide nanoparticles (nanospheres and nanorods). They used ZnO nanospheres with a diameter of 5 nm and nanorods with an average diameter 9 nm and length 60 nm, i.e., with aspect ratio of about 7. As a result, they demonstrated an increase in the NC absorption spectra compared to the azopolymer and ZnO nanoparticles absorption spectra.

A different technique was used for obtaining the NC thin films by Mateev et al. [185]. The nanocomposite thin films were prepared via the electrospray deposition of ZnO NPs with size in the range 40–100 nm over a thin azopolymer film. The dependence of the birefringence on the electrospraying time was determined. The optimal deposition time was 90 s. In this case, an increase in the birefringence of more than 25% compared to the film that was not electrosprayed was reached.

In other studies, nanocomposites are composed of photoanisotropic azo dyes, nanoparticles, and a non-photoanisotropic polymer matrix [168,169,170,171]. The chemical structures of some of the azo dyes used in these cases are shown in Figure 3.

#### 3.1.3. Silicon Dioxide (SiO_2_) Nanoparticles

Another type of nanoparticle commonly used for various optical applications is silicon dioxide, or silica (SiO_2_) NPs. In 2013, Nazarova et al. [144] prepared anisotropic organic/inorganic nanocomposite materials by incorporating 5–15 nm sized SiO_2_ NPs in a side-chain azopolymer. As a result, an enhancement of the photoinduced birefringence in these nanocomposite materials of about 20% compared to the non-doped sample was observed.

Yelleswarapu and Rao [186] developed nanoporous films in silica matrix with azobenzene molecules attached to the inner walls of these pores., This is the inverse type of nanocomposite compared to the ones we are considering. They demonstrated that these films are characterized by faster recording times and higher diffraction efficiency, compared to the conventional polymer films. The diffraction signal was twice as high in the case of 10 nm sized pores, and for the 50 nm sized ones, the diffraction signal increased five times. A similar “inverse” type of nanocomposite was reported by Reshetnyak et al. [187] in a study of the photo orientation of polymer fragments in a microporous glass.

The optical, chemical, and thermal properties of nanocomposite thin films based on azo dye methyl red (MR) hosted in polymethylmethacrylate (PMMA) with photo-initiator benzyl dimethyl ketal (BDK) and doped with 2.5% and 5% silica NPs were also investigated [143]. The authors confirm that PMMA–BDK–MR/silica NPs thin films could be successfully used in reversible optical data storage and UV-light sensors and reported increased thermal stability of the nanocomposite thin films.

#### 3.1.4. Titanium Dioxide (TiO_2_) Nanoparticles

A nanocomposite system was obtained by Kang et al. [145] by depositing Poly(Disperse-Red-19-p-phenyldiacrylate), i.e., PDR_19_ azopolymer into titanium dioxide, or titania (TiO_2_) nano porous films. The authors report improvement of the stability and efficiency of the polarization recording. 

In 2015, Fernandez et al. [146] studied a hybrid nanocomposite based on the azopolymer PAZO (here denoted as PCBS), TiO_2_ nanoparticles, and silver-containing TiO_2_ nanoparticles, generated via the sol–gel method. An enhancement of the thermal stability, glass transition temperature, and the induced birefringence was reached, but only for the case of the silver-containing hybrid nanocomposites.

Mateev et al. [147] investigated the photoinduced birefringence in azopolymer PAZO doped with spherical TiO_2_ nanoparticles with a size of 21 nm and different concentrations from 0 to 10 wt. %. An increase in the photoinduced birefringence was observed in the azopolymer films doped with TiO_2_ nanoparticles with a maximal value of ∆*n* for the sample with 1 wt. % concentration. Later research, reported in [188], revealed that the enhancement of the photoinduced birefringence can be further increased by thermal treatment.

#### 3.1.5. Semiconductor (Tellurium-Containing Chalcogenide System and Goethite) Nanoparticles

Stoilova et al. [149,164] studied NC films based on the azo polymer PAZO doped with 1 wt. % particles of the newly synthesized tellurium containing chalcogenide system (GeTe_4_)_100−x_Cu_x_, where x = 5, 10, 15 and 20 mol. %. An increase in the value of the photoinduced birefringence in comparison to the pure PAZO film has been observed for the sample doped with (GeTe_4_)_85_Cu_15_ particles.

Another type of semiconductor NP that has been studied as a dopant in azopolymers, is goethite (α-FeOOH), a characteristic antiferromagnetic material [129,150]. Goethite nanorods with size 15 × 150 nm, i.e., aspect ratio 1:10 were doped in PAZO with different concentrations, varying from 0% (undoped azopolymer film) to 15%. The nanocomposites thus obtained show a significant birefringence increase for the samples with 10% NPs concentration compared with the non-doped samples—nearly 70%. An unusual dependence of the birefringence and the diffraction efficiency on the concentration was observed—two peaks of enhancement at 1% and at 10% concentration—which we will discuss in Section 4.1, dedicated to the effect of nanoparticle shape.

#### 3.1.6. Zeolite and Other Nanoparticles

In 2014, Nazarova et al. [151] studied NPMs based on zeolite MFI (Mordenite Framework Inverted) NPs incorporated in azopolymers. Optical properties enhancement was observed in this material as well. Improvement of the photoresponse in thin films of these composite materials was observed compared to the non-doped samples—nearly 25% increase in the maximal value of the birefringence.

Earlier, Marlow and co-workers [189,190,191] investigated a similar combination between azopolymers and zeolites, but in a completely different configuration—zeolitic nanocomposites. They doped azo polymers in zeolite crystals and found that changes of the alignment of the guest molecules within the molecular sieve pores cause changes in the refractive index, or birefringence. They also described the irradiation wavelength dependence of the birefringence, the reversibility, and the stability of the photoinduced switching process.

In 2021, Liu et al. [152] synthesized NCs by doping upconverting nanoparticles (UCNPs) in a photoresponsive azobenzene-containing polymer. They used complex core-shell nanoparticles with hexagonal shape and obtained increased absorption. A possible application of this nanocomposite was proposed in the field of security.

#### 3.1.7. Birefringence Comparison for NPM Doped with Non-Metallic NPs

In order to compare the maximal birefringence value (Δ*n*_max_) for many samples with different NPs concentrations in the same matrix, the parameter “enhancement ratio” was introduced [144]. This parameter is defined as the maximal value of the birefringence for each sample divided to the same value for the non-doped sample: *E* = Δ*n*_max_(C)/Δ*n*_max_(0%). We use this parameter, because it is not influenced by the thickness of the specific sample or other properties of the experimental setup and shows only the change in the birefringence.

**Figure 4:**
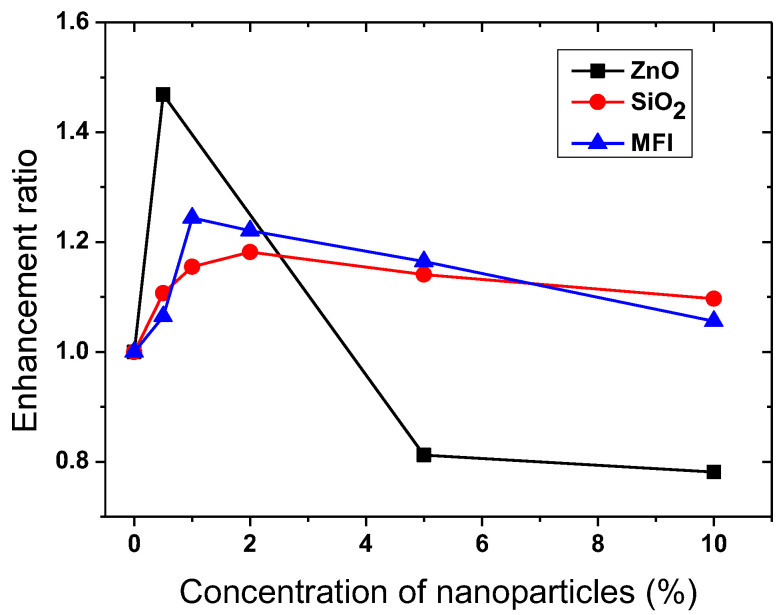
Dependence of the birefringence enhancement ratio on the concentration for different non-metallic NPs (ZnO, SiO_2_, MFI) in one and the same azopolymer matrix, namely the azopolymer P1 [139,144,151].

Thus, from the examined nanocomposite photoanisotropic materials, three types of nanoparticles incorporated in the same matrix (the azopolymer P1) were selected and the birefringence enhancement ratios versus concentration are presented in Figure 4.

It can be seen from the figure that the highest enhancement ratio for the same matrix was obtained for ZnO nanoparticles, followed by MFI and SiO_2_ NPs, with a significant increase observed for all types of NPs.

**Figure 5:**
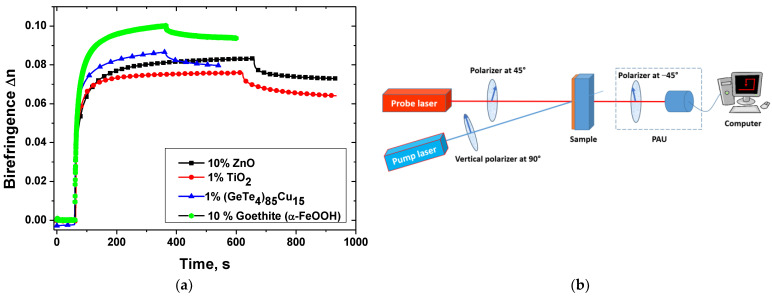
(**a**) Birefringence kinetics of PAZO matrix doped with different non-metallic NPs: ZnO [128], TiO_2_ [147], (GeTe_4_)_85_Cu_15_ [149], and goethite (α-FeOOH) [150] for their optimal concentrations; (**b**) optical scheme for birefringence kinetics measurement.

To illustrate the dynamics of the birefringence build-up, the birefringence kinetics for four types of nanoparticles (ZnO, TiO_2_, (GeTe_4_)_85_Cu_15_, and α-FeOOH) in the same PAZO matrix for their optimal concentrations are shown in Figure 5a.

A typical optical setup for photoinduced birefringence measurement is shown in Figure 5b. It consists of a pump laser with wavelength within the absorbance band of the nanocomposite material and with vertical polarization, a probe laser with wavelength not absorbed by the material and polarization of 45°, and a polarization analysis unit (PAU). This unit could represent either a polarizer oriented at –45° followed by a photodetector or a polarimeter. Measuring the intensity of the probe light transmitted through the crossed polarizers and the sample, or alternatively the Stokes parameters of light after the sample, allows for the obtention of the kinetics of birefringence in real time during the irradiation with the pump laser. In the case when “crossed polarizers” setup is used, the birefringence can be calculated as [114]:

(1) $$\Delta n=\frac{\lambda_{probe}}{\pi d}\arcsin\sqrt{\frac{I}{I_{0}}},$$

where *λ*_probe_ is the wavelength of the probe laser, *d* is the thickness of the thin film NPM sample, *I* is the intensity of the probe beam transmitted through the crossed polarizers and the sample, and *I*_0_ is the intensity of the probe beam passing through the polarizers oriented parallel to each other and the sample before the illumination with the pump laser.

When polarimeter is used, the birefringence is determined by the following expression [55]:

(2) $$\Delta n=\frac{\lambda_{probe}}{2\pi d}\arctan\left(\frac{S_{3}}{S_{2}}\right),$$

where *S*_2_ and *S*_3_ are two of the four Stokes parameters (*S*_0_, *S*_1_, *S*_2_, *S*_3_).

The experimental results for the birefringence kinetics for the optimal concentrations of the four types of NPs in Figure 5a were obtained following the same procedure. The first stage is to measure the background signal using only the probe laser. Then, the second stage is to irradiate the sample with linearly polarized light. Here, in the case of ZnO and goethite (α-FeOOH) NPs, the photoisomerization was performed with a laser with wavelength 473 nm. In the other two cases (TiO_2_ and (GeTe_4_)_85_Cu_15_ NPs), a 442 nm laser was used. After a certain time, saturation was reached and then the experiment continued with relaxation after the recording beam was stopped to estimate the stability of the recorded birefringence. As seen, the highest value of the photoinduced birefringence was obtained for the goethite nanoparticles.

### 3.2. NPM Doped with Metallic and Metallic Complexes Nanoparticles

Metallic NPs are suitable for fabrication of composite materials with novel optical properties since their optical response can be tuned by varying their size or shape. Metallic nanoparticles with different sizes and shapes are much easier to find than other nanoparticles. The optical properties of the metallic nanoparticles have been studied for decades [192]. The recently growing interest in their characteristics is due in part to the intensive development of nanocomposite materials. Metallic nanoparticles are made using different lithographic methods (nanosphere lithography [193,194], e-beam lithography [195], and others [196,197,198,199]) or variations of classical wet chemistry techniques. Lithographic methods produce well-defined sizes and shapes without aggregation and the other methods give non-spherical particles, especially rods [200,201] and triangles [202].

#### 3.2.1. Silver (Ag) Nanoparticles

In 2007, Zhou et al. [157] studied nanocomposite thin films containing azopolymer doped with spherical silver (Ag) nanoparticles. The influence of the concentration of the silver dopant on the reorientation of the azo groups was studied. An enhancement of about 50% for the reorientation rate and about 70% for the reorientation amplitude was achieved for the sample doped with 8.4 ng of Ag. In a later work from 2010, the authors observed a different trend for different substitutes of the azobenzene moieties [203]. They found that in the case of AzoCH3, photoorientation rate and birefringence increased with the increase in NP content, while in the case of AzoCN, these values had a minimum due to the cross-linking effect of the azopolymer around the NPs that restrained the mobility of the azobenzene molecules. They also obtained interesting results for the optically rewritable properties of the nanocomposites. Their results show that the stability of photoinduced birefringence of the nanocomposites is improved by introducing Ag nanoparticles into the azopolymer [155].

In 2015, Ledin et al. [165] reported the fabrication of silver nanocube arrays embedded in an active medium based on newly synthesized branched Azo-POSS compounds. They studied refractive index changes upon irradiation of the samples with UV light and repeatable shifts in silver nanocube LSPR peak position upon alternating irradiation of the composite thin films with UV and visible light.

In 2016, Hu et al. [170] synthesized Ag nanoparticles (with 5 nm average size) modified with azobenzene thiol and the enhanced light responsivity has been confirmed by two-photon induced optical data storage experiments.

Hautala in his MSc thesis also studied azobenzene-containing films doped with silver nanoparticles [161]. The polymer complex used was p4VP(DY7)_1.0_ obtained from the polymer poly(4-vinylpyridine) and the azochromophore Disperse Yellow 7. Silver/azopolymer mass ratios from 0 to 2% and up to 10% were studied. In general, he obtained an improvement in the diffraction efficiency and surface relief amplitude of the recorded polarization gratings.

Fernandez et al. [146] studied a hybrid nanocomposite based on the azopolymer PAZO and silver-containing TiO_2_ nanoparticles. They used the sol–gel method for synthesizing the nanoparticles. They achieved an enhancement of the thermal stability and glass transition temperature. Also, they obtained tenfold enhancement of the induced birefringence for the silver-containing hybrid nanocomposites compared to the non-doped samples.

Falcione et al. [132] in 2021 studied NCs containing PAZO and two different compositions of nanoparticles: Ag and AgAu (30% Ag/70% Au). The mass concentration of the NPs with respect to that of PAZO was 0, 0.03, 0.06, 0.09, and 0.12% for the samples with Ag NPs, and 0, 0.04, 0.08, 0.12, and 0.15% for the samples with AgAu NPs. The size of the nanoparticles was about 5 nm. For both types of NPs, they observed an enhancement of the optical properties of the nanocomposite materials. They determined that the optimal concentration for the AgAu NPs is 0.08% and for the Ag NPs is 0.09%. They performed a thorough study of the models proposed by different authors of the effect of the increased anisotropy of nanocomposites. Their results are explained in terms of the increased free volume for low concentrations and scattering for high concentrations of the nanoparticles.

#### 3.2.2. Gold (Au) Nanoparticles

In 2003, Manna et al. [204] synthesized and studied gold nanoparticles capped by an unsymmetrical azobenzene disulfide, 4-hexyl-4-(12-(dodecyldithio)dodecyloxy)azobenzene (C6AzSSC12), in order to investigate the efficiency of azobenzene photoisomerization on colloidal gold surfaces. The average size of the particles used is 5.2 nm. The authors studied the photoisomerization reaction of the C6AzSSC12-capped gold nanoparticles by UV-vis absorption spectroscopy in toluene and reported sedimentation of the C6AzSSC12-capped nanoparticles by photoisomerization from trans to cis isomers as evidence of the distinguishably higher efficiency of the photoreaction.

Later, in 2015, similar systems were investigated using differently substituted azobenzenes aimed at the precise control of the self-assembly of selected components within complex mixtures [205].

In 2005, Sidhaye et al. [206] also used functionalized with azobenzene derivatives Au NPs in order to show that the interparticle spacing in the networks could be controlled by the trans-cis and cis-trans isomerizations of the azobenzene moiety induced by UV and visible light, respectively.

In 2008, Na et al. [154] studied azoterpolymer (SE-AEC-MMA) films containing gold nanoparticles. The optimum concentration of the gold nanoparticles for the formation of SRG was found to be 0.06 wt. %. For this concentration, the SRG was about 2.2 times higher than that of the pure azopolymer film without gold nanoparticles. Their hypothesis about the mechanism responsible for this increase is that it is not only due to a field enhancement effect by the localized plasmon excitations of the gold nanoparticles, but is also a result of the chemical structure of the azoterpolymer used.

In the same year, Shin et al. [207] studied the photo response through change in the absorption spectra of azobenzene-alkanethiol functionalized gold nanoparticles.

In 2011, Zhang et al. [160] studied two-photon absorption and photoinduced anisotropy of a methyl orange/Au nanoparticles/polyvinylpyrrolidone (MO/Au/PVP) composite system. Au spherical nanoparticles with size 10–15 nm were used. Enhancement of the photoinduced birefringence of the MO film with Au nanoparticles was also observed. The birefringence of the pure film was 2.7 × 10^−3^ and it increased to 0.7 × 10^−2^ (or more than 2.5 times) for the NC film.

Yang et al. [159] in 2017 studied polarization holographic gratings recorded in similar nanocomposites, namely methyl orange/polyvinylpyrrolidone (MO/PVP) film doped with Au spherical nanoparticles with size 5–18 nm. Their results indicate that the diffraction efficiency of the gratings recorded in the NCs can increase 3.2 times compared with those inscribed in pure MO film. The maximum diffraction efficiency reached was 0.4%. Enhancement of the photoinduced birefringence of the NC samples was also observed. The birefringence of the non-doped film was 9.8 × 10^−4^ and it increased to 1.6 × 10^−3^ for the NC film.

In 2019, our group also started a series of experimental studies on nanocomposites containing gold nanoparticles doped in PAZO [156]. Initially, spherical Au NPs with 10 nm average size were used. Samples with five concentrations of the NPs varying from 0 to 4 a.u. (1 a.u. = 0.015 wt. %) were prepared. An increase in the photoinduced birefringence for pump laser with wavelength 442 nm in doped azopolymer nanocomposite films compared to samples of undoped azopolymers was observed. The maximal enhancement was achieved for 2 a.u. concentration of the Au NPs. Later, in order to study the effect of the size of the nanoparticles, nanocomposites with the same polymer and gold nanoparticles with an average size of 10 nm, 20 nm, 30 nm, 40 nm, and 50 nm were also investigated [158]. An increase in birefringence and stability and a decrease in the response time of the materials were obtained. These studies were performed at six different NP concentrations—0, 1, 2, 3, 4, and 16 a.u. A 25% increase in the birefringence for the case of 20 nm sized Au NP with concentration C = 2 a.u. was demonstrated. Polarization gratings with two spatial frequencies were recorded using two recording angles and a diffraction efficiency of more than 28% was reached [158]. The influence of the Au NP size on the parameters of the photoinduced birefringence, diffraction efficiency, and surface relief of the polarization holographic gratings recorded in this azopolymer nanocomposite were also investigated and are discussed in Section 4.2. and Section 5. 

One of the investigated model systems is azobenzene-modified gold surfaces (AMGS). In 2009, Klajn et al. [208] used Au NP (5.6 nm diameter) or Ag NP (5.3 nm diameter) inks coated with mixed self-assembled monolayers of dodecylamine and photoswitchable azobenzene-terminated thiol (4-(11-mercaptoundecanoxy)azobenzene (MUA) to demonstrate the opportunity and flexibility of non-equilibrium nanostructures to create materials capable of changing their properties or functions on demand in response to external stimuli.

In 2022, Kunfi et al. [209] studied the light-induced and thermal properties of an AMGS system of azobenzenes on morphologically different PDA/Au substrates. Azobenzene derivatives having different chain-lengths were used and attached to the prepared surfaces. The thermal isomerization was accelerated for particle-bound azobenzenes compared to those in solution.

In 2019, Sunaga et al. [210] reported the synthesis, characterization, and chiral optical properties of azo-group-containing chiral Schiff base metal complexes absorbed on gold nanoparticles of 10 nm diameters by polarized UV light irradiation.

Elhani et al. [153] in 2020 studied DR1/Au-NPs/PMMA nanocomposite thin films with 0.3, 0.5, 0.7, and 2 vol. % concentration of spherical Au NPs with size 15 nm. Maximal enhancement of the absorption was obtained for 0.7% concentration of the NPs. They reported that all NPs concentrations lead to absorption enhancement of DR1 in PMMA and both the absorption and photo-orientation were enhanced.

Very recently, in 2023, Loșmanschii et al. [211] reported a study on NC materials containing Au NPs with diameter of 25 nm and concentrations of 0, 0.0004, 0.0006, and 0.001 mg/mL incorporated in poly-n-epoxypropilcarbazole (PEPC) with Solvent Yellow 3(SY3) azopolymer. The influence of the Au NPs concentration on the absorption coefficient, the refractive index, the bandgap, and the magnitude of the photoinduced anisotropy of the nanocomposites was evaluated.

#### 3.2.3. Cooper (Cu) and Nickel (Ni) Metallic Complexes

The study of photoanisotropic nanocomposites has been extended with another dopant: bioactive metal (Cu, Ni) complexes [162,163]. Copper is considered to be an antioxidant trace element in human cells acting as a cofactor in a number of redox enzymes. It is an essential element, required for normal brain development and functioning. The transition metal nickel is an indispensable component of several metalloenzymes involved in energy and nitrogen metabolism. In the study of Mateev et al. [162], enhanced photoinduced birefringence was reported in composite films of the azopolymer PAZO, doped with metal complexes of Cu(II) 3-amino-5,5′-dimethylhydantoin (CLP) and Ni(II) 3-amino-5,5′-dimethylhydantoin (NLP) with different concentrations. Significant enhancement in the value of the maximal photoinduced birefringence has been observed for the hybrid materials in comparison to a pure PAZO polymer film. A maximal photoinduced birefringence of 0.093 has been reached for the composite film with 1 wt. % particles of the Ni(II) 3-amino-5,5′-dimethylhydantoin. The corresponding value of the non-doped PAZO polymer thin film is 0.080. Amongst the NCs with particles of the Cu(II) 3-amino-5,5′-dimethylhydantoin, the highest birefringence was found for the composite film doped with 2 wt. % CLP.

#### 3.2.4. Birefringence Comparison for NPM Doped with Metallic and Metallic Complex NPs

**Figure 6:**
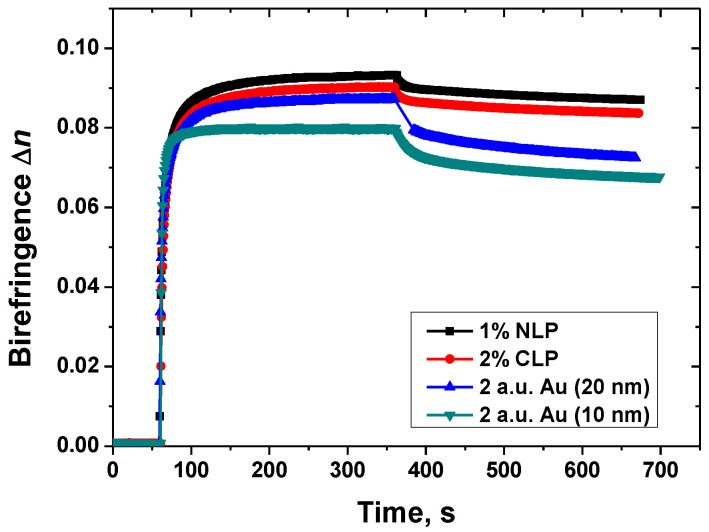
Birefringence kinetics of the azopolymer PAZO doped with different metallic NPs: Au [156,158], Ni, and Cu complexes [162,163] for their optimal concentrations.

Similar to Figure 5, but for metallic NPs, the birefringence kinetics for some nanoparticles in the same PAZO matrix for their optimal concentrations are shown in Figure 6.

As mentioned earlier, the birefringence measurement consists of three stages: (i) the background signal assessment; (ii) photoisomerization until saturation is reached to determine the maximal birefringence value, and finally (iii) relaxation, aimed to estimate the stability of the birefringence in time. As seen, the highest value of the photoinduced birefringence was obtained for the Ni-ion nanoparticles.

The data about the main optical parameters of the various nanocomposites presented in this Section are summarized and compared in tabular form in Section 6.

## 4. Nanocomposite Photoanisotropic Materials Doped with Nanoparticles with Different Shapes and Sizes

### 4.1. NPM Doped with Nanoparticles with Different Shapes

Nanorods have demonstrated extremely promising properties for optical applications both at the single nanoparticle level and for large assemblies [212,213]. The two very attractive properties of nanorods, namely anisotropic shape and high surface-to-volume ratio, make them desirable nanomaterials for many applications, like photovoltaics, sensors, and building blocks for the construction of logic elements and memory circuits [214,215,216,217,218]. The physical properties of elongated inorganic nanoparticles are extensively explored in the review article of Krahne et al. [212]. They demonstrate that the shape anisotropy of nanorods leads to several interesting features in their optical behavior, such as linearly polarized emission, tunable emission wavelength, and large absorption cross sections paired with strong quantum confinement. These properties make nanorods promising and more interesting than spherical nanoparticles in many aspects for implementation into device architectures.

The first study of the dependence of birefringence on the shape of nanoparticles in NCs containing an azopolymer doped with nanorods was done by Nedelchev et al. in 2016 [150]. As we mentioned earlier, in this work the photoinduced birefringence in nanocomposite films of an azopolymer (PAZO) doped with goethite nanorods was investigated. The NPs have elongated shape with size 15 × 150 nm i.e., ratio 1:10. Concentration of the NPs was varied from 0% to 15%. Two maxima of the birefringence, for the samples with 1% and 10% NP concentration, were observed. This is an unusual dependence of the birefringence on the concentration having two peaks of enhancement.

Previous studies with ZnO, SiO_2_ and zeolite MFI NP have indicated only one peak of increase in this dependence. A hypothesis is proposed that this effect could be related with the elongated shape of the nanoparticles and the presence of two characteristic NP sizes—15 and 150 nm. In the case of MFI nanoparticles [151], the nanocrystals are rectangular with sizes about 15 × 25 nm, i.e., aspect ratio 1:1.7 and exhibit almost plate-like morphology. The small aspect ratio in this case does not lead to two distinguishable peaks in the dependence of the birefringence on the nanoparticle concentration. In contrast, for larger aspect ratios, as in the case of goethite nanoparticles, two peaks are clearly observed in this dependence.

Later, in a second work with the same nanocomposite (azopolymer PAZO doped with goethite nanorods), polarization holographic gratings were recorded and studied [129]. Increased diffraction efficiencies and surface relief were obtained, confirming the birefringence results. The dependence of the diffraction efficiency on the concentration of nanorods in the nanocomposite has the same two-peak pattern as that of the birefringence (Table 1).

Thus, as it is seen from Table 1 and works [129,150], we can conclude, that for spherical particles with one characteristic size, the dependence of birefringence on the concentration of nanoparticles has one peak, and for nanorods with two characteristic sizes, the same dependence has two peaks.

In 2017, Cao et al. [219] investigated the dependence of the optical properties on the shape of the nanoparticles comparing the diffraction efficiency in nanocomposites doped with either gold nanospheres or gold nanorods. They used Au nanorods with an average diameter of 10 nm and an average length of 34 nm as dopants in phenanthrenequinone-doped poly(methyl methacrylate) photopolymers and obtained diffraction efficiency 29.6% higher than that in the pure photopolymer and 18.5% higher than that with spherical dopants. 

In [152] Liu et al. used core-shell nanoparticles with hexagonal shape, but they oriented their work more towards the application of the resulting nanocomposites and there are no data on the shape dependence of the birefringence to be used in this analysis. 

Similar is the case with the study of silver nanocubes from Ledin et al. [165]. They studied refractive index changes and local surface plasmon resonances of these nanocomposites and there is no data on the shape dependence of the birefringence.

In the work of Shah et al., where nanospheres and nanorods were studied, photoisomerization of azo NCs was found to be less efficient in the case of nanorods as compared to spherical nanoparticles [142]. In that case, however, the nanocomposite consists of azobenzene compound grafted on the surface of nanorods or nanospheres of ZnO. They explain this result with the fact that the behavior of the elongated nanohybrids resembles that classically obtained on flat substrates where long-range packing forces are strong. As a result, only a small number of accessible azo chromophores can reach the *cis* state. Indeed, the extent of packing is dependent on the shape of the nanoparticle and nanorods, with flat facets, enabling a tighter organization of the molecules in the self-assembled monolayer than in the case of nanodots that display a more curved shape. Consistently, the efficiency of photochromic switching has also shown to be strongly affected by nanoparticle shape. The comparison of which particles are more efficient in this case can be made only in the case of NCs with NPs matrix and azo dopant and in the reverse case with Azo matrix and NPs dopant this consideration cannot be applied.

### 4.2. NPM Doped with Nanoparticles with Different Sizes

In 2013, the first study on the effect of nanoparticle size in photoanisotropic nanocomposites on the optical properties of the materials was done by Nazarova et al. [166]. In order to determine the influence of the size of nanoparticles, two major parameters that characterize the response of any photoanisotropic media have been studied, namely the maximal value of the photoinduced linear birefringence, and the response time. Series of nanocomposite films based on two amorphous azopolymers P_1_ and P_1–2_, doped with ZnO nanoparticles with two different sizes—50 nm and 100 nm were used for this study. It was found that the nanocomposite layers doped with both 50 and 100 nm NP have up to 30% faster response compared with the non-doped layers. Doping with the smaller (50 nm size) NPs leads also to a significant increase in more than 40% in the maximal value of the photoinduced birefringence. On the other hand, doping with the larger (100 nm) NPs leads to a decrease in this parameter.

In Hautala’s study mentioned above, three different sizes of silver nanoparticles, namely 8 nm, 30 nm and 50 nm, were used [161]. Different silver/azopolymer mass ratios were used for the NPs with sizes 30 and 50 nm (ranging from 0 to 2%) and for the 8 nm NPs mass ratios were up to 10%. Hautala obtained an improvement in the optical properties only for the 8 nm Ag particles. In contrast, for the 30 nm and 50 nm sized NPs no increase in the diffraction efficiency and surface relief was observed.

A study on the influence of the size of the doped particles in NCs on their photoanisotropic properties with the largest size variation was done in 2021 by the research group of Nazarova and Nedelchev and was reported by Berberova et al. [158]. Nanocomposites containing the azopolymer PAZO and gold nanoparticles with five different average sizes—10 nm, 20 nm, 30 nm, 40 nm and 50 nm were investigated. For each of the NP sizes, thin film samples with six different concentrations (0, 1, 2, 3, 4 and 16 a.u.) were prepared and characterized. Polarimetric studies were performed to determine the maximum birefringence, as well as polarization holographic recording and AFM studies to determine the diffraction efficiency and the height of the resulting surface relief. 

An increase in birefringence, diffraction efficiency, surface relief, stability and a decrease in the response time of the NC materials were obtained. The enhancement was most pronounced for concentration *C* = 2 a.u. and NP size 20 nm and to some extent for the 10 nm sized NPs. Significant increase in the birefringence with 25% for the case of 20 nm sized Au NPs and 15% for 10 nm Au NPs was demonstrated. These results indicate that the optimal diameter of the Au NPs leading to highest increase in the photoinduced birefringence is 20 nm at least for the given azopolymer and pump laser wavelength 442 nm. A detailed discussion on the mechanism of enhancement in NPM is given in Section 6. Again in Section 6, in tabular form we provide a summary and comparison between the main optical parameters of the various nanocomposites discussed in this Section.

## 5. Surface Relief Gratings Recorded in NPM—Impact of the Nanoparticles on the Surface Relief Formation

A very small part of the research groups working with nanocomposite photoanisotropic materials presented in this review, have investigated the formation of surface relief gratings in these materials during polarization holographic recording. Examples of such groups are Na et al. for NPM with Au NPs [154], our group for NPMs with ZnO, TiO_2_, bioactive metal complexes with Cu and Ni, goethite (α-FeOOH) nanorods and Au [129,141,148,158,163], Achimova et al. with QDs [135], Falcione et al. with Ag and AgAu NPs [132], and Hautala with Ag NPs [161].

**Figure 7:**
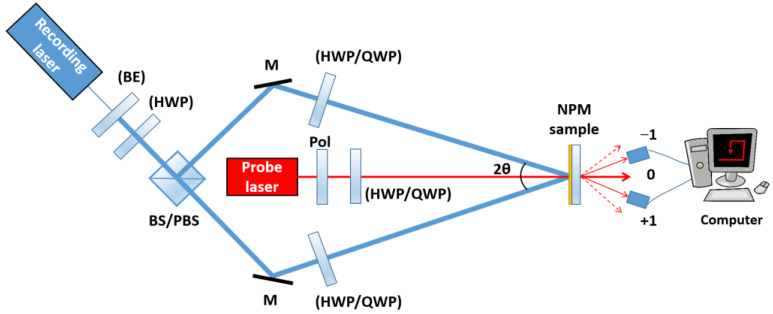
Optical scheme for polarization holographic and surface relief grating recording.

A generalized optical scheme for polarization holographic and surface relief grating recording is presented in Figure 7. The recording laser is a coherent laser with wavelength within the absorbance band of the NPM used, and with sufficient intensity. The probe laser is typically a low-intensity laser with wavelength which is not absorbed by the NPM sample, most commonly a He-Ne laser (633 nm) or DPSS laser at 635 nm. In addition to the recording and the probe laser, the setup includes beam expander (BE), beam splitter or polarization beam splitter (BS/PBS), polarizer (Pol), half-wave plates (HWP) and quarter-wave plates (QWP) for the wavelengths of recording/probe laser. The elements whose symbols are enclosed in brackets on the scheme are optional. In the simplest case, using only polarization beam splitter and no waveplates, the polarizations of the recording beams will be horizontal and vertical (or *s-p*). Any other desired polarizations of the recording beams, for example linear at ±45°, left and right circular, etc., can be obtained by suitable settings of the HWP/QWP. The value of the angle between the recording beams (2*θ*) determines the period *Λ* of the recorded grating, as follows:

(3) $$\Lambda =\frac{\lambda_{\mathrm{rec}}}{2\sin\theta }$$

where *λ*_rec_ is the recording laser wavelength and *θ* is half of the recording angle 2*θ*.

Na et al. first studied the effect of the gold nanoparticles on the formation of SRG in azoterpolymer films containing Au NPs [154]. They found that the height of the surface relief gratings is strongly dependent on the content of gold nanoparticles dispersed in the azopolymer layers. The optimum concentration of gold nanoparticles for the formation of SRG was found to be 0.06 wt. %. In this case the height of SRG was about 100 nm. This is 2.2 times higher than in the case of non-doped azopolymer film.

The effect of ZnO nanoparticles on the surface relief formation in polarization diffraction gratings in photoanisotropic nanocomposite materials was first studied in 2017 [141]. The nanocomposite material used was based on the azopolymer PAZO with incorporated ZnO nanoparticles with size < 50 nm. Polarization holographic gratings with two spatial frequencies (625 lines/mm and 1250 lines/mm) were obtained and comparison of the parameters of gratings recorded in NC materials and pure azopolymer was made. The experimental results indicated that both the diffraction efficiency and the height of the surface relief for the nanocomposite samples were enhanced. The height of the relief was increased with about 50% for spatial frequency 625 lines/mm and 3 times for spatial frequency 1250 lines/mm. The diffraction efficiency was also enhanced with 20% for the 625 lines/mm grating and twice for the 1250 lines/mm grating.

Later, in nanocomposites with the same matrix (PAZO) but doped with TiO_2_ NPs, polarization holographic gratings were studied [148]. A maximal diffraction efficiency of 27.2% for 3 wt. % concentration of TiO_2_ NPs for the samples deposited from methanol solution was achieved. The gratings with maximal diffraction efficiency from the two series of experiments with samples coated from water and methanol solutions also have the highest surface relief of 275 nm and 530 nm respectively. An enhancement of about 40% of the height of the surface relief for the nanocomposite samples was achieved.

A similar study on the formation of stable in time surface relief in azopolymer films doped with particles of the transition biometals nickel and copper complexed by aminohydantoin ligands was reported in [163]. Significant enhancement of the surface relief height and diffraction efficiency has been observed for the NC materials in comparison to the pure PAZO polymer films. Diffraction efficiency of 33.0% and SRG height of 586 nm have been reached for the composite film with 1 wt. % particles of the Ni(II) 3-amino-5,5′-dimethylhydantoin. For the NCs with particles of the Cu(II) 3-amino-5,5′-dimethylhydantoin highest diffraction efficiency was 31.1%, and highest profile height 557 nm, for the composite film doped with 2 wt. % NPs. The highest surface reliefs among those considered in this review were obtained in these nanocomposite materials.

Surface relief gratings with periods from 0.86 µm to 2.51 µm with relief modulation close to 300 nm were inscribed in nanocomposite samples composed of azopolymer and goethite nanorods [129]. The diffraction efficiency in the sample with optimal concentration of the goethite nanorods reaches nearly 27%. The dependence of the diffraction efficiency on the concentration of nanorods in the nanocomposite had the same two-peak pattern as that of the birefringence—one peak at low concentrations (0.5–2 wt. %) and one at higher concentrations (10 wt. %). The highest relief of 280 nm was observed for the grating with highest diffraction efficiency, which was inscribed in the sample with 10% NPs concentration.

Polarization gratings with two grating periods (or spatial frequencies) of 1.3 μm (or spatial frequency 790 lines/mm) and 0.85 μm (or 1200 lines/mm) have been recorded using two recording angles in NCs with Au NPs in PAZO by Berberova et al. [158]. Diffraction efficiency greater than 28% was obtained for the sample containing 10 nm Au NP along with surface relief height of 360 nm. 

Using nanocomposites based on the azo copolymer PEPC-co-DO doped with QDs, Achimova et al. [135] reported an increase in the diffraction efficiency in the nanocomposite materials compared to the pure polymer, but the height of the surface relief gratings recorded in the pure polymer films was higher.

Falcione et al. studied NCs of PAZO and two different types of nanoparticles: Ag and AgAu (30% Ag/70% Au) [132]. For both types of NPs, they observed that diffraction efficiency can be optimized. For the holographic recording a Lloyd’s Mirror interferometer optical set up was used, with incident angle corresponding to a fringe period of about 2.7 μm. They found that for some concentrations of NPs (0.08% for AgAu NPs and 0.09% for Ag NPs) the surface relief modulation and diffraction efficiency were higher than these obtained for PAZO films without NPs. Surface relief modulations of 320 nm and 400 nm were reached for 0.08% AgAu NPs and 0.09% Ag NPs, respectively. They proposed a simple model for the diffraction efficiency as a function of the induced surface relief height. Like Berberova et al. [141], and other reports for surface relief formation in NCs [129,148,158,161,163], they also observed that the increase in the diffraction efficiency is related to the increase in the surface relief modulation.

As it was mentioned before, Hautala in his investigations on NCs containing the azopolymer p4VP(DY7) doped with Ag nanoparticles also performed holographic recording [161]. He obtained an improvement of the diffraction efficiency and surface relief of the recorded holographic gratings in the case of 8 nm Ag NPs. The diffraction efficiency of the recorded polarization gratings increased from 2.4% to 4.2%, while surface relief from 90 nm to 110 nm. For 30 nm and 50 nm NPs no increase in diffraction efficiency and surface relief was observed.

**Figure 8:**
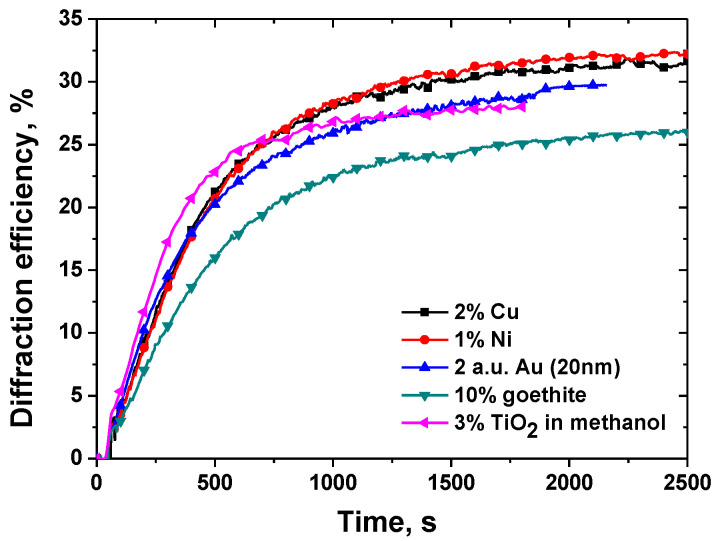
Diffraction efficiencies kinetics of thin films of PAZO doped with different NPs: ZnO [141], TiO_2_ [148], α-FeOOH [129], Au [158], Ni and Cu complexes [162,163] for their optimal concentrations.

A comparison of the diffraction efficiencies achieved during polarization holographic recording for some of the nanocomposites described in this review is shown in Figure 8. Amongst the presented nanocomposites, the highest efficiency was obtained for those containing Ni complexes.

**Figure 9:**
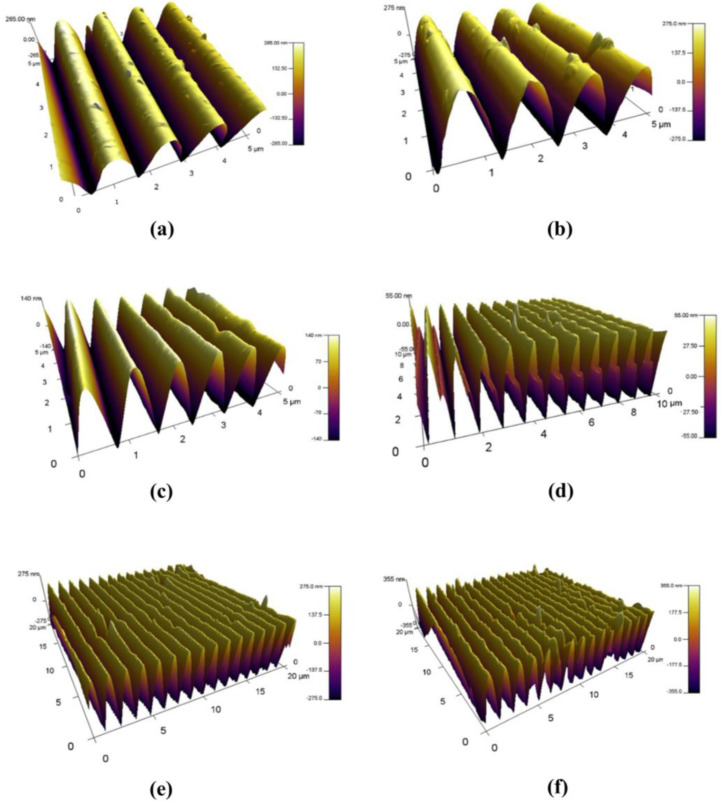
Three-dimensional AFM images of the surface relief gratings, formed in nanocomposite materials during polarization holographic recording. In all cases the azopolymer used was PAZO. The dopant NPs concentrations and types are: (**a**) 2% Cu complexes; (**b**) 1% Ni complexes; (**c**) 10% goethite nanorods; (**d**) 16 a.u. Au NPs with size 40 nm; (**e**) 1% TiO_2_ NPs with size 21 nm; (**f**) 3% TiO_2_ NPs with size 21 nm.

Some examples of surface relief gratings formed during polarization holographic recording in nanocomposite photoanisotropic materials are shown in Figure 9. They are obtained using atomic force microscopy (AFM).

The data about the diffraction efficiency and height of the surface relief of the different NPM presented and discussed in this Section, are summarized and compared in tabular form in the following Section.

## 6. Summary and Discussion

Finally, we summarize all the data about the photoanisotropic nanocomposites presented in this review, their components and characteristics, as well as the obtained results, in Table 2. In addition to the size, shape, and composition of the doped nanoparticles and the nanocomposite matrices, the obtained increases in birefringence, diffraction efficiency, and relief height of the recorded diffraction gratings as well as the corresponding reference are also presented in the table. Not all authors investigated each of these parameters and therefore they are presented where available.

As can be seen from the table and as we mentioned in the beginning of this review, there are some preferred or most often used components for the nanocomposite photoanisotropic materials. The most often used non-metallic nanoparticles are ZnO and CNT and among the metallic nanoparticles, the most often used are Ag and Au. The most frequently used azopolymer matrix is the commercially available PAZO, and among azodyes the most frequently used are Methyl Orange (MO) and Disperse Red 1 (DR1).

Most of the authors declare an increase in the photoanisotropic properties of the materials as a consequence of doping with nanoparticles. In some cases, decrease has also been observed due to the specific method used for the synthesis of nanoparticles. Such, for example, is the case of the polymers doped with CNT and CNF via the mixing method compared to the in situ doped polymers in the work of Rodríguez-González et al. [137]. In other cases, the optical, chemical, and thermal properties like absorption, reversible isomerization, rewriting stability, thermal stability, and others were investigated [142,143,152,155,209].

From the analysis of Table 2, it can be seen that an increase in photoinduced birefringence has been reported by many researchers, with the largest increase in over 40% obtained in [129,137,139,146,150,157,159,160]. For comparison and evaluation of the results, however, the value of the maximum achieved birefringence is more important. From this point of view, the highest maximum birefringence values of 0.1; 0.097; 0.093; and 0.09 were achieved in [129,149,150,155,162,163], respectively.

A similar analysis can be applied to the diffraction efficiencies and heights of the surface relief obtained when recording diffraction gratings in the nanocomposite materials. Diffraction efficiency values of 36%; 33.8%; 33%; 31.1%; 28.6%; and 26.9% were reached in [129,132,135,158,163], respectively. The studied photoanisotropic nanocomposites were used to record diffraction gratings and surface relief heights of 586 nm; 557 nm; 530 nm; 400 nm; 391 nm; and 360 nm were achieved [132,135,148,158,163].

However, it is important not only *how much* the various optical recording characteristics increase in nanocomposite materials, but also *why* they increase, or in other words, what is the mechanism responsible for this optical properties’ enhancement. When we study the processes in the various composite systems and the reasons for their better optical performance, we find significant differences in the NCs used for conventional holography and those used for polarization holography. In conventional holography, the processes are more straightforward because the main enhancement in nanocomposites is caused by the displacement of the nanoparticles into either the bright or the dark holographic interference fringes, depending on the type of the matrix and the nanoparticles. As a consequence of this rearrangement, higher differences between the refractive indices in these bands are obtained, which in turn lead to higher diffraction efficiency of the holographic recording [12].

In polarization holography, however, there is no intensity modulation, only polarization modulation. Thus, displacement of the nanoparticles is not expected and will not affect the processes. Therefore, the improvement of the photoanisotropic properties of nanocomposite over undoped anisotropic materials is expected to be due to other processes.

To date, three main hypotheses for mechanisms have been investigated and reported, which possibly explain why doping with NPs leads to an increase in birefringence and a subsequent increase in the diffraction efficiency and the height of the resulting surface relief of the recorded diffraction gratings in the nanocomposites composed of azopolymers and nanoparticles. These hypotheses are the following:

1. **Free volume hypothesis**: the addition of NPs increases the free volume around the azo chromophores and hence improves the processes of photoisomerization, rotation and reorientation, making both the rate of photoisomerization and the number of molecules available for isomerization higher [132,139,144,147,158,167,188].
2. **Scattering hypothesis:** The addition of NPs increases the scattering inside the NC. Because the scattering is in all directions, the scattering of light by the NPs would allow for the excitation and reorientation of the originally off-plane chromophores that would otherwise not contribute to the birefringence [132,139,144].
3. **Localized surface plasmon polariton resonance (LSPPR) hypothesis**: In the case of metal NPs, the hypothesis that the increase in birefringence is due to the existence of localized surface plasmon polariton resonance has often been proposed. In fact, light interaction with NPs at the LSPPR frequencies produces electromagnetic fields around the NPs many orders of magnitude higher than the incident fields and exciting the plasmon resonance of the particles would lead to light absorption and local heating, which clearly makes it easier for the molecules to reorient [153,154,155].

In most studies regarding azopolymer/metallic NPs nanocomposites, results that showed increased values for the photo-orientation rate, the maximum birefringence, or other optical recording characteristics, were explained by the effect of LSPPR [153,154,155,157,203]. Indeed, at the plasmon resonances, the field enhancement and the temperature increase in the vicinity of the NPs would favor the azobenzene mobility and enhance the photoisomerization rate. 

Zhou et al. observed a maximum of the photo-orientation rate and the birefringence for an amount of Ag NPs of 10 ng [157]. Measurements performed at different wavelengths allowed to infer phenomenologically that LSPPR in Ag NPs immersed in the polymer matrix would be the key factor for the improvement of the optical properties. The observed enhancement of the photo-orientation of the azo dyes under photo-selective polarized light irradiation in the study of Elhani et al. is also explained by the enhanced absorption of the azo dyes in the presence of the plasmonic nanoparticles that act as nanoantennas for surface enhanced visible light absorption [153].

Localized surface plasmon polariton resonance hypothesis, however, has one major drawback: this hypothesis cannot explain the increase in the photoanisotropic properties of nanocomposites doped with non-metallic nanoparticles reported by many authors. Such are the results of Nedelchev et al. [139] and Shah et al. [142] in 2012 for ZnO NPs, Nazarova et al. [144] in 2013 and Alsaad et al. [143] in 2021 with SiO_2_ NPs, Mateev et al. [147,188] and Kang et al. [145] with TiO_2_ NPs and many others listed in the section “Non-metallic nanoparticles” of Table 2.

In 2011, Vijayakumar et al. [167] suggested that more volume for the photoisomerization of azobenzene was provided by wrapping of the polymer chains around SWCNT surface.

In 2012 [139] and 2013 [144], the group of Nazarova and Nedelchev suggested two possible mechanisms, corresponding to hypothesis 1 and 2 described above, which may explain the observed enhancement. The fact that the *trans–cis* isomerization of azobenzene chromophores requires a free volume is reported as early as 2006 by Dall’Agnol et al. [220]. In [139] it was suggested that NPs might reduce the interaction between the azobenzene molecules close to the surface of the NPs resulting in enhanced mobility and higher birefringence, or in other words NPs are used as spacers of the polymer chains. It is also clear that free volume increases with the concentration due to the higher contact surface between the azo molecules and the NPs. All this leads to increase in the birefringence and this process depends only on the size of the NP, but not on their chemical composition or refractive index, because smaller size results in larger surface/volume ratio. Another possible mechanism that can assists the reorientation of the azomolecules in presence of NP was suggested in [144]. It is related to the scattering from NPs and the “off-plane” chromophores that are oriented perpendicularly to the sample plane and are not influenced by the recording polarized light. The light scattered from the NP, though, has components in all directions and so it can be absorbed also by this “off-plane” azochromophores. In such a way they can contribute to the increase in the birefringence. After extensive investigations on NPM containing different NPs, in 2021 our group succeeded to study the dependence of the optical response of nanocomposites on the size of the nanoparticles using Au NPs with different sizes [158]. The obtained results indicate that the increase in birefringence and surface relief height is more significant for smaller nanoparticles, namely for Au NP with 20 nm diameter. The scattering effect however, should be stronger for larger nanoparticles. This leads to the conclusion that the enhancement is due to a larger extent to the free volume effect than to the scattering from the nanoparticles. This is also confirmed by an earlier report when only two sizes of ZnO were studied in [164].

The hypothesis of the free volume as a main factor for the improved optical properties of NCs has also been confirmed by the use of NCs as membranes [221], as well as in the study of the sensing properties of NCs with incorporated Ag NPs [222]. NPs prevent the polymer chain from packing close and increase the free space in the polymer matrix and facilitate the movement of the polymer chains [221,222]. In [223] the authors optimized their azopolymer material in order to improve the photoanisotropy for optical memories storage applications by balancing the free volume with the azobenzene concentration.

Then it is reasonable to ask why the process does not increase indefinitely with the increase in NPs concentration, but instead an optimal NP concentration is observed in the reports of the scientific groups working in the field. As noted by Falcione et al. [132], a 5 nm diameter NP would increase the separation between two polymer chains because the distance between them is about the length of the azo chromophore (~1 nm) [224]. With this argument in mind, it follows that the higher the concentration of NPs, the greater will be the free volume in the material. As the NPs concentration increases, the free volume increases and there are more azo groups available to isomerize, which leads to an increase in birefringence and a subsequent increase in the diffraction efficiency and the height of the resulting surface relief of the recorded diffraction gratings in the NPM. However, when the concentration of NPs exceeds certain value, the process of enhancement of the photoanisotropic properties of the NPM begins to reverse. NPs begin to occupy more space per unit volume, reducing the number of azo groups in this volume. So, it turns out that there is a certain concentration, different for each type of NPs, which is optimal for the desired properties of NPM. This mechanism, describing the influence of the NPs on the photoanisotropic properties of NPM, is in accordance with the experimental results, reported by Vijayakumar et al. [167], Nedelchev et al. [129], Nazarova et al. [144,148,156,166], Falcione et al. [132] and Berberova et al. [141,158].

## 7. Photoanisotropic Nanocomposites: Future Outlook

The wide variety of applications indicated above shows unequivocally that photoanisotropic nanocomposites have already made the transition from the laboratory to use in the advanced technologies. Photoanisotropic nanocomposites can be obtained by almost infinite combinations of materials: various photoanisotropic matrices and nanoparticles with different compositions, sizes, concentrations, shapes and morphology. Although the application possibilities are many and varied, only a limited number of these material combinations as constituents of nanocomposites have been explored, as seen in the selected studies shown in Table 2.

In addition, more progress could be made towards optimizing nanocomposites for their applications in polarization holographic recording and surface relief formation. Regarding the practical production and implementation of photoanisotropic nanocomposites for real applications, these materials are still in their infancy. Some particular research areas offer significant opportunity for future developments, such as the use of composite nanoparticles, or core-shell nanoparticles, or NPs with more exotic forms for doping in photoanisotropic matrices, the ability to enhance or control thermal emission or coupling of light with thermal, electrical or other processes, and exploring the long-term behavior of nanoparticles in real nanocomposite systems. Furthermore, many researchers are already interested in exploring the possibilities to dynamically control the optical properties of nanoparticles and hence nanocomposites through various mechanisms. This is expected to lead to new approaches to control a wide range of processes in the photoanisotropic nanocomposites that have not yet been explored.

## 8. Conclusions

Photoanisotropic nanocomposites based on an azodye or azopolymer matrix with added nanoparticles are an attractive and relatively easy to produce materials with improved optical characteristics. Due to the wide variety of simple synthesis techniques that can be used to obtain nanoparticles of different compositions, shapes and sizes, the optical properties of nanocomposites can be precisely tuned and adjusted for specific applications.

Most of the results presented in Table 2 show an increase in one or all of the parameters: birefringence, diffraction efficiency, or surface relief height of the polarization holographic gratings recorded in the nanocomposites compared to the undoped material. Other optical parameters, such as absorption, response time, stability, etc., were also investigated. Nanocomposites with different compositions were studied, varying both the type of nanoparticles and the type of matrix. Both metallic and non-metallic particles have been studied, with gold and silver nanoparticles being the most commonly used metallic ones, and zinc oxide and quantum dots being the most frequently used non-metallic ones. The preferred photoanisotropic matrix amongst the azopolymers is the commercially available polymer PAZO, and amongst the azodyes, the most common are Methyl Orange and Disperse Red 1.

The optimal concentrations of the nanoparticles were determined, as well as in some cases their optimal size. The influence of the shape of the nanoparticles on the optical properties of the nanocomposites was also investigated.

Nanocomposite materials, unlike other optical materials, have a much wider range of applications due to their composite nature. One of their components, namely nanoparticles, can be implemented in different ways; for example, they can be dispersed in a liquid or applied as a thin film. This opens up the possibility of a wide range of potential applications. New opportunities are being discovered for the emergence of new products that exploit the unique optical advantages provided by nanocomposite materials.

## Figures and Tables

**Table 1:** Relation between the nanoparticle shape and type of birefringence dependence on nanoparticles concentration.

	Nanosphere	Nanorod
Shape	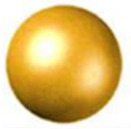	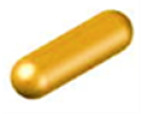
Birefringence dependance on concentration	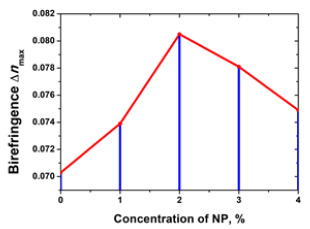	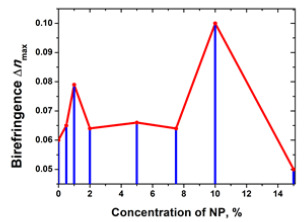

**Table 2:** Summary of the nanocomposites’ components and the main optical characteristics in case of polarization recording: birefringence, diffraction efficiency, and surface relief height.

No.	NP Type	NP Size	NP Shape	NP Concentration, Optimal	Matrix: Polymer, Copolymer or Dye	Birefringence Increase/Max Value	DE Increase/Max Value	SR Height Increase/Max Value	Refs.
**Non-metallic nanoparticles: QDs, CNT, CNF, oxides, semiconductors**									
1	QDs CdS366 CdS433 CdS441	2.43 nm 4.57 nm 4.89 nm			DR-1	Enhancement of global absorbance			[136]
2	chalcogenide QDs Cd_0.2_Zn_0.8_Se	3.5 nm	Sphere	1.5%	PEPC-co-DO	-	1.24 to 1.28%	No increase	[134,135]
3	CNT	140 nm	Tubes	0.5%	P0C6 and M0C6	0.068 to 0.087 28%	-	-	[137]
4	CNF		Fibers	0.5%	P0C6 and M0C6	0.068 to 0.097 42%	-	-	[137]
5	CNT	1.5 μm/20–40 nm	Tubes	0.0005 g in 0.0025 g DO3	Poly(lactic acid) (PLA) and Disperse Orange 3 (DO3)	Enhancement of the degree of photoinduced optical anisotropy in a wide temperature range			[138]
6	SWCNT	μm/2–50 nm	Tubes	0.2–0.4 mg/mL	P1,P2,P3 azobenzene/pyrene (1/1; 1/1; 0.7/0.3)	0.015 to 0.018	The thermal stability increased		[167]
7	SWCNT	1.5 nm/2 μm	Tubes	0.42, 0.56%	PMMA/Azo chromophore	Switching amplitudes up to 28% were achieved			[168]
8	MWCNT	20–30 nm/15 μm	Tubes	0.5%	PMMA/Azo benzene chromophore	Switching amplitudes up to 10% were achieved			[169]
9	ZnO	<50 nm	Sphere	0.5%	P1, P2, P1-2	47%, 19%, 41%, to 0.082	-	-	[139]
10	ZnO	<100 nm	Sphere	0.5%	P1, P1-2	decrease			[166]
11	ZnO	5 nm 9/60 nm	Sphere, nanorods		AZO	Absorption studied			[142]
12	ZnO	<50 nm	Sphere	10%	PAZO	34%	20%, twice	9 nm; 42 nm 625 lines/mm; 1250 lines/mm	[128,141]
13	ZnO	60–100 nm	sphere	90 s deposition	PAZO	20%	-	-	[140]
14	SiO_2_			2.5% and 5%	PMMA–BDK–MR	Studied the optical, chemical, and thermal properties: absorption and the thermal stability			[143]
15	SiO_2_	5–15 nm	Sphere		P1	20%	-	-	[144]
16	TiO_2_	Nanoporous film	Nanoporous film	PDR19/TiO_2_ 3%	PDR19	-	0.04 to 0.14%	-	[145]
17	TiO_2_	21 nm	Sphere	3%	PAZO/methanol	0.0757–0.0795 5%	25.1–27.2%	337–530 nm	[148]
18	TiO_2_	21 nm	Sphere	1%	PAZO/water	0.0725–0.076 5%	10.5–12.6%	120–275 nm	[147,148]
19	TiO_2_ SG	Sol–gel (SG)	Sol–gel (SG)	10–99,67%	(PCBS) or PAZO	Absorption and reversible isomerization studied			[146]
20	α-FeOOH	15/150 nm	Nanorods	0, 0.5, 1 *, 2, 5, 7.5, **10**, 15%	PAZO	0.065 to 0.1 54%	18.5% to 26.9%	170 to 280 nm	[129,150]
21	(GeTe_4_)_100−x_ Cux	50–100 nm	Sphere	1%	PAZO	0.07 to 0.09 29%	-	-	[149]
22	MFI	15 × 25 nm	Plate-like morphology	1%	P1	25%	-	-	[151]
23	Upconverting nanoparticles (UCNPs)	50 nm 57 nm	Hexagons	10% 20%	PAzo	Absorption studied and anticounterfeiting applications			[152]
**Metallic nanoparticles and metallic complexes**									
24	Ag	10 nm	Sphere	8.4 ng	Copolym	50%	-	-	[157]
25	Ag	10 nm	Sphere	2.2, 3.3, 4.5, **5.6**%	Poly(AzoCN-co-HEMA (442;514 nm)	Increased rewriting stability			[155]
26	Ag	5 nm	Sphere	-	Azo-SH/PMMA	Absorption studied			[170]
27	Ag	8 nm	Sphere	0.5–1.5%	p4VP(DY7)1.0	-	2.4 to 4.2%	90–110 nm	[161]
28	Ag	30 nm	Sphere	0–2%	p4VP(DY7)1.0	-	decrease	decrease	[161]
29	Ag	50 nm	Sphere	0–2%	p4VP(DY7)1.0	-	decrease	decrease	[161]
30	Ag	5 nm		0, 0.03, 0.06, **0.09**, 0.12%,	PAZO	-	36% >2x	225 to 400 nm	[132]
31	Ag-Au (30%Ag/70% Au)	5 nm		0, 0.04, **0.08**, 0.12, 0.15%	PAZO	-	33% >2x	225 to 325 nm	[132]
32	Ti/Ag SG	sol–gel (SG)	Sol–gel (SG)	10–99.67%	(PCBS) or PAZO	10 times	-	-	[146]
33	Au	5.2 nm	Sphere	-	C6AzSSC12	Absorption and sedimentation studied			[204]
34	Au	-	-	0.03, **0.06,** 0.1, 0.13%	SE-AEC-MMA azoterpolymer	-	-	45–100 nm 2.2x	[154]
35	Au	15 nm		0.2–2% **0.7%**	DR1/Au-NPs/PMMA	-	2.5x	-	[153]
36	Au	10 nm	Sphere	0, 0.015, **0.03**, 0.045, 0.06, 0.24%	PAZO	0.07–0.08 14%	25.4–28.6%	330–360 nm	[156,158]
37	Au	20 nm	Sphere	0, 0.015, **0.03**, 0.045, 0.06, 0.24%	PAZO	0.07–0.087 24%	25.4–26.8%	-	[158]
38	Au	30 nm	Sphere	0, 0.015, 0.03, 0.045, 0.06, **0.24**%	PAZO	0.07–0.077 1%	25.4–26.2%	-	[158]
39	Au	40 nm	Sphere	0, **0.015**, **0.03**, 0.045, 0.06, **0.24**%	PAZO	0.07–0.074 5%	-	330–340 nm	[158]
40	Au	10–15 nm	Sphere	2.91 × 10^−4^ M	MO/polyvinylpyrrolidone (PVP)	0.0027 to 0.007 or 2.6x	-	-	[160]
41	Au	5–18 nm	Sphere	-	MO/polyvinylpyrrolidone (PVP)	9.8 × 10^−4^ to 1.6 × 10^−3^ 63%	0.12–0.42% 3.2x	-	[159]
42	Au	25 nm	Sphere	0, 0.0004, 0.0006, **0.001** mg/mL	PEPC-co-SY3	Photoinduced changes in absorption, azimuth, and ellipticity			[211]
43	PDA/Au Polydopamine/Au	30–50 nm 30–80 nm 600–900 nm			Azobenzene derivatives with different spacer lengths AzoC2SH, AzoC3SH, AzoC6SH	The thermal isomerization was accelerated for particle-bound azobenzenes compared to those in solution			[209]
44	Ni(II) 3-amino- 5,5′-dimethylhydantoin (NLP)			**1**, 2, 5%	PAZO	0.08–0.093 16%	24–33%	321–586 nm	[162,163]
45	Cu(II) 3-amino-5,5′-dimethylhydantoin (CLP)			1, **2**, 5%	PAZO	0.08–0.091 14%	24–31.1%	321–557 nm	[162,163]

* The optimal concentrations are marked with bold.

## Data Availability

All data generated or analyzed during this study are included in the published article.
